# Identification of a conserved neutralizing epitope in Seneca Valley virus VP2 protein: new insight for epitope vaccine designment

**DOI:** 10.1186/s12985-022-01791-5

**Published:** 2022-04-11

**Authors:** Wei Wen, Xinghua Chen, Qiang Lv, Huanchun Chen, Ping Qian, Xiangmin Li

**Affiliations:** 1 State Key Laboratory of Agricultural Microbiology, Huazhong Agricultural University, Wuhan, 430070 Hubei Province People’s Republic of China; 2 College of Veterinary Medicine, Huazhong Agricultural University, Wuhan, 430070 Hubei People’s Republic of China; 3 The Cooperative Innovation Center for Sustainable Pig Production, Wuhan, 430070 Hubei People’s Republic of China; 4 Key Laboratory of Development of Veterinary Diagnostic Products, Ministry of Agriculture of the People’s Republic of China, Wuhan, 430070 Hubei People’s Republic of China

**Keywords:** Seneca Valley virus, Monoclonal antibody, Neutralization test, VP2 protein, Neutralizing epitope

## Abstract

**Background:**

Seneca Valley virus (SVV) is a picornavirus that causes vesicular disease in swine. Clinical characteristics of the disease are similar to common viral diseases such as foot-and-mouth disease virus, porcine vesicular disease virus, and vesicular stomatitis virus, which can cause vesicles in the nose or hoof of pigs. Therefore, developing tools for detecting SVV infection is critical and urgent.

**Methods:**

The neutralizing antibodies were produced to detect the neutralizing epitope.

**Results:**

Five SVV neutralizing monoclonal antibodies (mAb), named 2C8, 3E4, 4C3, 6D7, and 7C11, were generated by immunizing mouses with ultra-purified SVV-LNSY01-2017. All five monoclonal antibodies exhibited high neutralizing titers to SVV. The epitopes targeted by these mAbs were further identified by peptide scanning using GST fusion peptides. The peptide ^153^QELNEE^158^ is defined as the smallest linear neutralizing epitope. The antibodies showed no reactivity to VP2 single mutants E157A. Furthermore, the antibodies showed no neutralizing activity with the recombinant virus (SVV-E157A).

**Conclusions:**

The five monoclonal antibodies and identified epitopes may contribute to further research on the structure and function of VP2 and the development of diagnostic methods for detecting different SVV strains. Additionally, the epitope recognized by monoclonal antibodies against VP2 protein may provide insights for novel SVV vaccines and oncolytic viruses development.

## Background

Seneca Valley virus (SVV) is a positive single-stranded RNA virus belonging to the *Picornaviridae* [1]. In the early stage of infection, the virus colonizes the tonsils and begins to replicate and multiply in large quantities, and then enters the bloodstream to cause viremia, which passes through the blood circulation and enters the main organs [2, 3]. And in the early stage of the disease, the sick pigs showed anorexia, lameness, fever, and snooze; then the skin or mucous membranes such as the nose, tongue, and hooves formed white swelling, followed by ulceration [3–5]. The first strain of Seneca Valley virus (SVV-001) was isolated in 2002 [6]. It was used to treat certain neuroendocrine tumors, such as non-small cell lung cancer [7]. Therefore, it has always been considered a non-pathogenic virus. Since 2015, a large number of SVV infections has been observed in piglets in Brazil [8], the United States [9], and China [10, 11]. The veterinary diagnostic laboratory at Iowa State University in the United States has sequenced the whole gene and found that the homology of all newly isolated strains is as high as 99%-100%. However, the difference was significant compared to the early SVV (1988–2011) sequence [12]. Phylogenetic tree analysis showed that SVV has mutated in the past 30 years with increased pathogenicity.

SVV virion is an icosahedral structure with no capsule and a diameter of 27 nm. The viral genome contains 7280 nucleotides [13]. SVV genome contains a single open reading frame (ORF) encoded by 6543 nt, which is cleaved into four structural proteins and eight non-structural proteins [14]. The P1 polypeptide is cleaved by 3C protease into VP0, VP3, and VP1, which constitute the viral nucleocapsid, and the mature VP0 is cleaved to form VP2 and VP4 [1]. VP1, VP2, and VP3 proteins are distributed on the outer surface of the capsid, while VP4 protein is on the inner surface of the capsid [1, 15]. Previous studies have shown that VP1, VP2, and VP3 proteins can induce the production of neutralizing antibodies. The antigenicity is relatively strong and conservative, and the proteins can be considered the main diagnostic target antigen of SVV [16–19].

To promote a comprehensive study of SVV infection and antiviral molecular mechanisms, we have prepared a series of neutralizing monoclonal antibodies (mAbs) against SVV VP2 protein in this study. The function of these monoclonal antibodies has been verified by neutralization assay, indirect enzyme-linked immunosorbent assay (iELISA), indirect immunofluorescence assay (IFA), and western blot. The epitope recognized by monoclonal antibodies against VP2 protein may provide new insight for vaccine development against SVV virulent strains.

## Methods

### Reagents and materials

SVV-LNSY01-2017 used in this study was isolated from the pig with Porcine primary vesicular disease (PIVD). Baby hamster kidney cells (BHK21 cells; ATCC, CCL-10) were cultured in Dulbecco’s modified essential medium (HyClone, SH30022.01) containing 10% fetal bovine serum (Gibco, 16000044) and 100 U/ml of penicillin (GENVIEW, GA3502) at 37 °C in a humidified 5% CO2 incubator. Myeloma cells SP2/0 (ATCC, CRL-1581) were grown in RPMI 1640 medium (HyClone, SH30809.01) containing 10% FBS, 100 U/ml of penicillin grown under the same conditions as those described above. SPF level BalB/c mice were purchased from the Animal Experimental Center of Huazhong Agricultural University. Protein A/G Agarose (sc-2003) was purchased from Santa Cruz Biotechnology. Alexa Fluor 555 goat anti-mouse (A32727) antibodies were obtained from Invitrogen. Polyvinylidene fluoride (PVDF, 46978100) membranes were purchased from Roche.

### Virus propagation and purification

BHK-21 cells were infected with an amount of 0.01 MOI SVV-LNSY01-2017 when the cell culture area reached 80%. BHK-21 cells with typical cytopathic effects (CPE) were collected at 48 h post-infection (hpi) and then repeatedly frozen and thawed 2 times, and then the virus solution was harvested. The virus solution was concentrated and precipitated at 30,000 rotating speed per minute (r/min), and then the supernatant was discarded and the precipitation was resuspended with PBS (pH = 7.4). The precipitation was dissolved at 4 °C for 12 h, and then the supernatant was collected by centrifugation at 12,000 rpm and then centrifuged at 40,000 rpm by a sucrose gradient. Subsequently, the virus band was taken up for de-sucrose treatment, and the precipitation was resuspended with PBS (pH = 7.4) and stored at − 80 °C. Purified viruses were further identified by SDS-PAGE and western blot analysis.

### Monoclonal antibody production

4-week-old female SPF BalB/c mice purchased from the Laboratory Animal Center of Huazhong Agricultural University (Wuhan, China), were subcutaneously immunized with 40 µg of purified SVV-LNSY01-2017 particles with an equal amount of complete Freund's adjuvant. After the initial immunization, mice were immunized 2 times with the same dose of purified SVV-LNSY01-2017 particles with an equal amount of incomplete Freund's adjuvant at two-week intervals. After mice were immunized three times, serum samples were collected from the immunized mice and serum titers were detected by indirect ELISA/virus neutralization assay. Three days after the fourth booster immunization, mice splenocytes were harvested and fused with SP2/0 using 50% polyethylene glycol (50% PEG, Sigma, Aldrich). Hybridoma culture supernatants were screened by virus neutralization assay and the positive hybridoma cells were cloned by a limiting dilution. Stable hybridoma clones (10^6^ cells) were injected intraperitoneally into liquid paraffin-pretreated abdominal cavities of BalB/c mice. Then the monoclonal antibodies were harvested and purified by a Protein A/G purification kit (NAbTMProtein A/G Spin Kit, Thermo Scientific, Fremont, CA, USA), and their activity was characterized by western blot and indirect immunofluorescence assay (IFA).

### Virus neutralization assay

The antibody-secreting hybridoma cells are screened by virus neutralization assay. Briefly, the hybridoma cell supernatant was serially diluted twofold, mixed with 200 TCID_50_ SVV-LNSY01-2017 in equal volumes, and placed in a 37 °C CO_2_ incubator for 2 h. The mixture was inoculated into a single layer of BHK-21 cells in a 96-well plate at 100 μL/well and cultured for 3 to 4 days in a CO_2_ incubator at 37 °C. Then the cytopathic condition was recorded to identify neutralizing hybridoma cells. The neutralizing hybridoma cells were subcloned into new 96-well plates by limiting dilution. After three rounds of subcloning, the hybridoma cells capable of stably secreting neutralizing antibodies were obtained.

### Indirect enzyme-linked immunosorbent assay

ELISA plates were coated overnight at 4 °C with 100 μL of inactivated SVV-LNSY01-2017 (1 μg/mL) diluted in bicarbonate coating buffer (1.59 g/L Na_2_CO3 and 2.93 g/L NaHCO3, pH = 9.6). The wells of the plate were washed 3 times with PBS and then blocked with 5.0% bovine serum albumin (BSA) in PBS (PBSA) for 2 h at 37 °C. The wells were drained and incubated with 100 μL of two-fold serially diluted mAbs (from 1:100 to 1:12,800) for 1 h at 37 °C. The wells were then washed three times with PBS containing 0.05% Tween-20 (PBST) and then incubated with 100 μL of horseradish peroxidase (HRP)-conjugated goat anti-mouse IgG (1:10,000, Boster, Wuhan, China) for 1 h at 37 °C. After 3 times washing with PBST, the reactions were developed with 50 μL/well substrate A (0.1 M citrate/phosphate buffer [pH 5.0]) and 50 μL/well solution B (0.04% o-phenylenediamine; 0.14% H_2_O_2_) for 10 min at room temperature and then terminated with 50 μL/well of 2 M H_2_SO_4_. The optical densities (OD) at 630 nm were measured using a microplate reader.

### Western blot identification of monoclonal antibodies

293 T cells were transfected with plasmids to express the indicated protein. At 24 h posttransfection, cells were harvested and treated with lysis buffer (1.19% HEPES, 0.88% NaCl, 0.04% EDTA, 1% NP-40) containing a protease inhibitor (Roche, UK), and then incubated on ice for 30 min. Equal amounts of proteins were subjected to 12% sodium dodecyl sulfate polyacrylamide gel electrophoresis (SDS-PAGE) and then the separated protein was transferred onto polyvinylidene fluoride (PVDF) membranes (Roche, UK). The membrane was blocked with 5% skim milk at room temperature for 2 h, and then the membrane was incubated with the indicated antibodies for 2 h at room temperature. The membrane was washed 5 times with PBST, and then incubated with horseradish peroxidase (HRP)-labeled goat anti-mouse IgG (1:5000) for 1 h. The positive bands were visualized by electrochemiluminescence (ECL) reagents and developed on film.

### Indirect immunofluorescence identification

BHK-21 cells were cultured in 96-well plates. When the cells are full of a single layer, the cells were inoculated with 2000 TCID_50_ SVV-LNSY01-2017. When the cell cytopathic effect reached 70–80%, it was fixed with 4% paraformaldehyde for 30 min and permeated with 0.2% Triton X-100 at room temperature for 20 min. The cells were blocked with PBS (pH = 7.4) containing 3% BSA for 30 min and then washed with PBS three times. 100 μL/well of hybridoma cell supernatant was added to different culture wells and the samples were incubated at 37℃ for 1 h. After the cells were washed with PBS three times, Alexa Fluor 555 goat anti-mouse antibodies (1:1000 dilution) were added and the samples were incubated for 30 min in dark at room temperature. The results were observed under a fluorescence microscope.

### Construction of mutant infectious SVV cDNA clones

Infectious SVV cDNA clones were conserved in our laboratory. Mutagenesis of SVV-E157A construct was performed using site-directed mutagenesis (C214-01/02; Vazyme). The construct was identified through DNA sequencing. To rescue viruses, plasmids containing full-length viral cDNAs were transfected into 293 T cells using JetPRIME (PT-114–07; Polyplus Transfection) according to the manufacturer’s protocol. When about 70% of cells exhibited CPE, the media was collected. Then the virus was serially passaged five times on BHK-21 cells. Stocks from each passage were stored at -80˚C. Each passage viruses were sequenced to identify the stability of the virus.

### Statistical analysis

The preceding experiments were performed in triplicate. The various treatments were compared by an unpaired, two-tailed Student's t-test while assuming unequal variance. *P* < 0.05 was considered statistically significant. Meanwhile, *P* < 0.001 and *P* < 0.0001 were marked with two (**) and three (***) asterisks, respectively.

## Results

### Preparation of immunogen

**Fig. 1:**
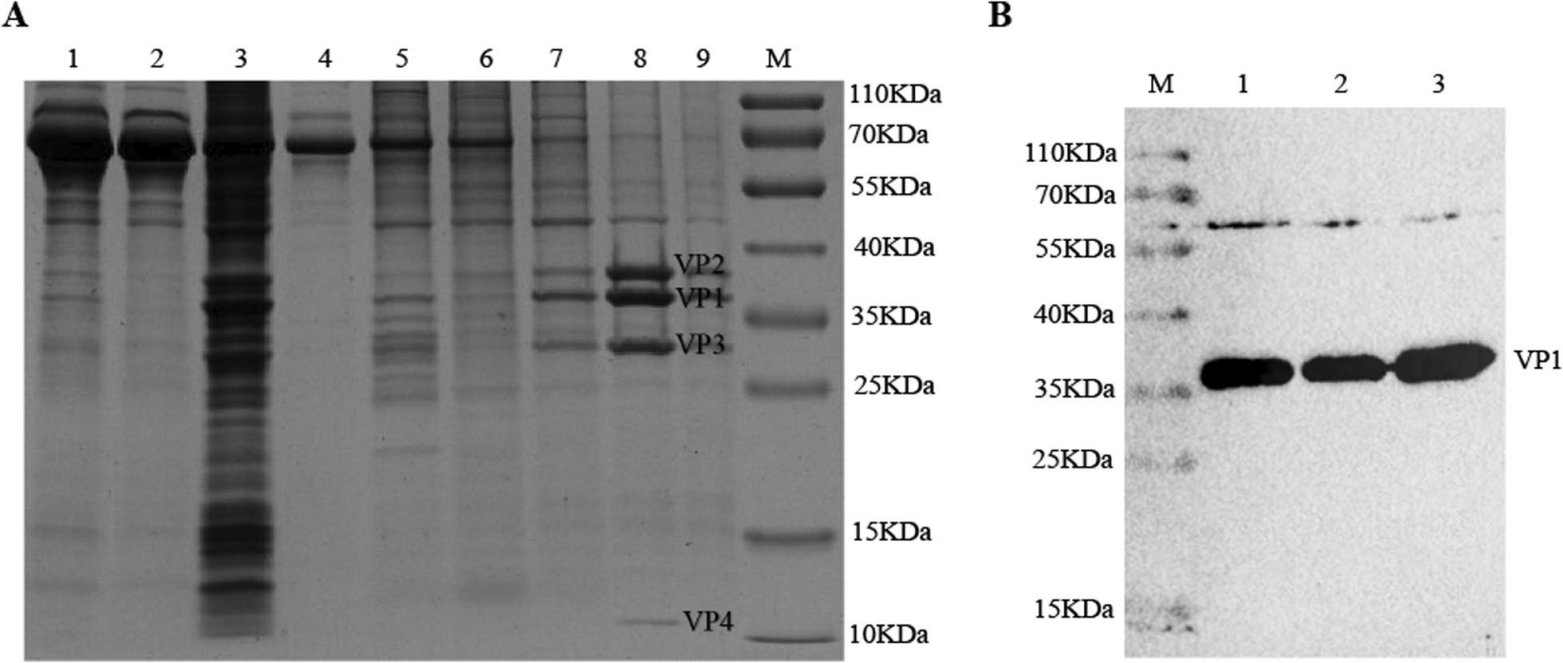
Immunogen preparation. **A** Components of the samples during ultra-high speed centrifugation were analyzed by SDS-PAGE and visualized by Coomassie blue staining. 1: Clear cell debris; 2: Concentrated supernatant; 3: Concentrated precipitate; 4: Sample layer; 5: 20% sucrose layer; 6: 35% sucrose layer; 7: 50% sucrose layer; 8: Virus Bands layer; 9: 65% sucrose layer; M: Protein Marker. **B** After the virus was desucrosed, the rabbit-derived antibody against SVV VP1 protein prepared in our laboratory was used as a primary antibody, and the purified SVV was identified by western blot. M: Protein Marker

To obtain large batches of viruses, BHK-21 cells were infected with 0.01 MOI SVV. When the CPE occurred, the virus solution was collected after the cells were frozen and thawed. Viral bands were obtained by ultra-high-speed centrifugation and sucrose gradient centrifugation, and the purified virus was confirmed by SDS-PAGE. As shown in Fig. 1A, SVV structural proteins VP1, VP2, VP3, and VP4 were correct in size, and impurity protein was less in lane 8. Western blot analysis was performed using SVV VP1 antibodies. The result showed that VP1 protein was detected and the size was as expected (Fig. 1B). The result demonstrated that we obtained high purity and high concentration of SVV particles, and the purified virus can be used to immunize mice to manufacture mAbs.

### Functional analysis of mAbs

**Fig. 2:**
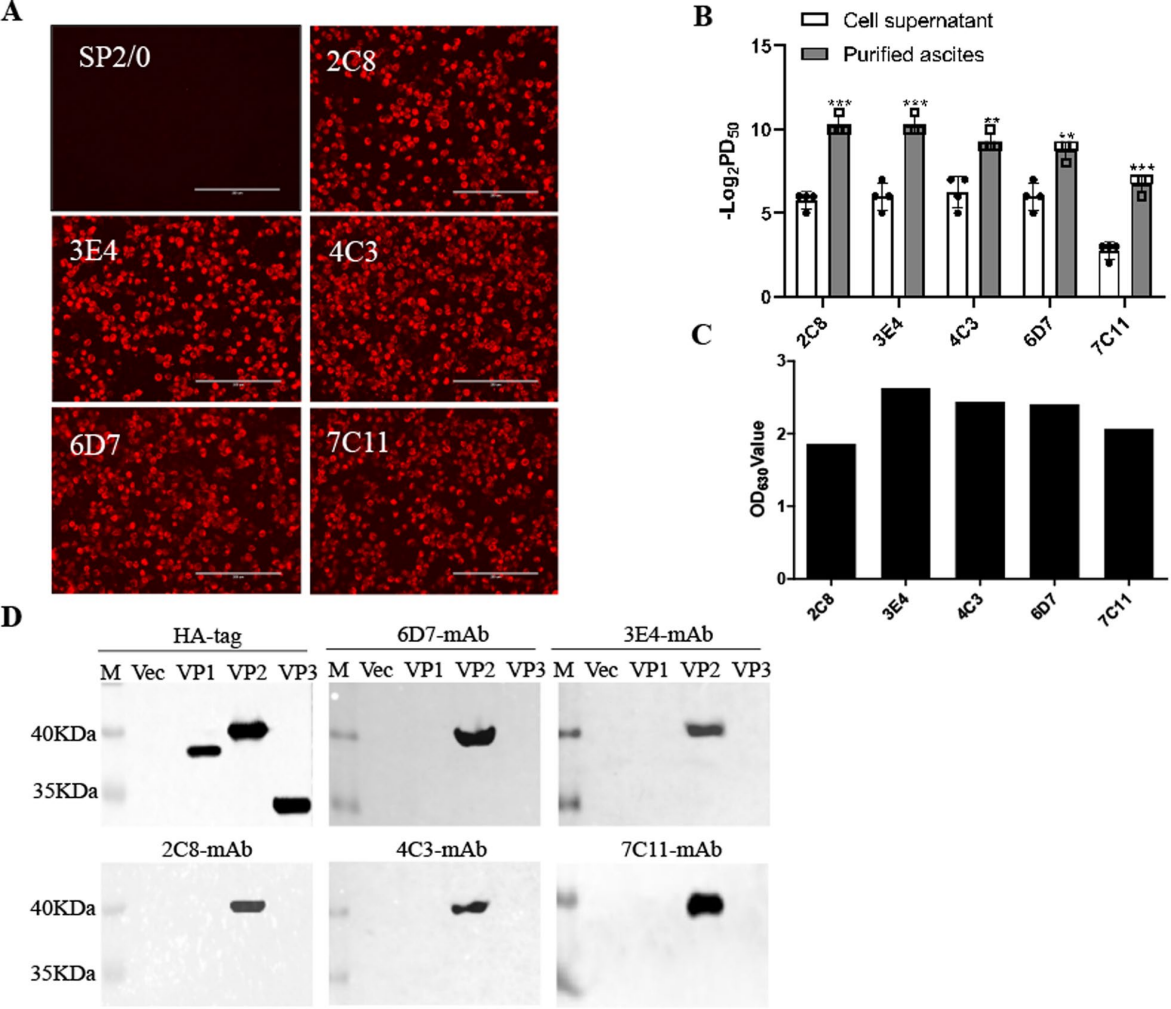
Functional analysis of monoclonal antibodies. **A** BHK-21 cells were infected with 0.01 MOI SVV-LNSY01-2017 for 36 h and then conducted for immunostaining analysis with 5 monoclonal antibodies. The mouse SP2/0 cell culture supernatant was used as a negative control. The cells were analyzed by an inverted fluorescence microscope. Scale bar: 200 μm. **B** The hybridoma cell supernatant and purified ascites of the five monoclonal antibodies were twofold multiple dilution and mixed with 200 TCID_50_ SVV. BHK-21 cells were incubated with virus-antibody mixtures to determine their neutralizing titers. **C** The prepared five monoclonal antibodies were used as primary antibodies in an indirect ELISA to test their reactivity with the virus. **D** 293 T cells were transfected with plasmids encoding VP1, VP2, or VP3. The cell lysates were conducted for western blot analysis with 5 monoclonal antibodies

To verify whether these monoclonal antibodies are suitable for IFA measurement, a single layer of BHK-21 cells was infected with 0.01 MOI SVV. After cytopathic effect (CPE) occurred, the cells were stained with the prepared five monoclonal antibodies. As shown in Fig. 2A, the cells staining with monoclonal antibodies displayed bright red fluorescence signals while the cells staining with SP2/0 cultured supernatant did not, indicating that all the antibodies can react well with viral proteins. Subsequently, we performed neutralizing titer tests of these monoclonal antibodies. BHK-21 cells were incubated with virus-antibody mixtures and cultured for 3 days. The Reed-Muench method was used to calculate the neutralization titer. The results showed that the ascites neutralization titers of the five antibodies were significantly higher than those of the culture supernatant, and the ascites neutralization titers of 2C8 and 3E4 could reach 1: 2^10^ (Fig. 2B). Next, we tested the application of these prepared mAbs in ELISA. The readout of the ELISA plate demonstrated that all five mAbs can be used as primary antibodies to detect SVV-LNSY01-2017 by using the ELISA assay (Fig. 2C). Western blot analysis showed that all mAbs can react with VP2, but not VP1 or VP3 (Fig. 2D). Collectively, these results indicate that five neutralizing antibodies react with SVV VP2.

### Epitope mapping of VP2 protein

**Fig. 3:**
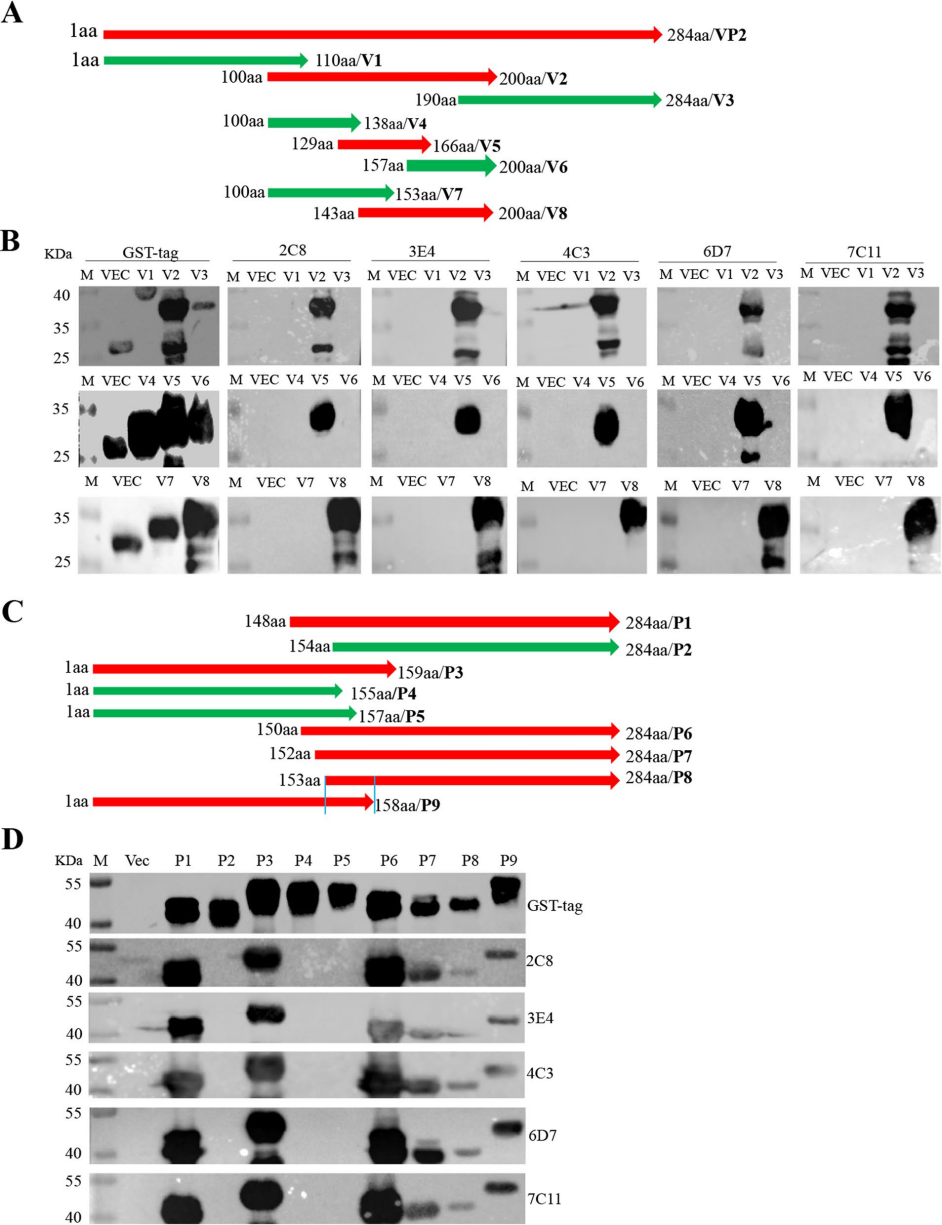
Identification of VP2 protein neutralizing linear epitopes by the antibodies. **A** Schematic diagram of the SVV VP2 protein fragment used for epitope mapping. V1: 1-110aa; V2: 100-200aa; V3: 190-284aa; V4: 100-138aa; V5: 129-166aa; V6: 157-200aa; V7: 100-153aa; V8: 143-200aa. **B** 293 T cells were transfected with plasmids encoding VP2 truncations for 24 h. The cell lysates were conducted for western blot analysis with the antibodies. **C** Schematic diagram of the SVV VP2 protein fragment used for epitope mapping. P1: 148-284aa; P2: 154-284aa; P3: 1-159aa; P4: 1-155aa; P5: 1-157aa; P6: 150-284aa; P7: 152-284aa; P8: 153-284aa; P9: 1-158aa. **D** 293 T cells were transfected with plasmids encoding VP2 truncations for 24 h, and then the cell lysates were conducted for western blot analysis with the antibodies

To determine the linear epitopes recognized by these prepared monoclonal antibodies, the full length of SVV VP2 gene was truncated to 8 parts, with 20 bp overlap between adjacent parts (Fig. 3A), and the truncations were constructed into the pGEX-KG vector for expression of eight truncated proteins. Western blot analysis showed that all eight truncated proteins were successfully expressed in BL21 (DE3) E.coli. Strikingly, all five monoclonal antibodies recognized fragments containing the region 143-166aa (Fig. 3B). To determine the fine epitopes of these mAbs, nine truncations between the 143-166aa region were constructed (Fig. 3C). The results showed that all five antibodies recognized the fragment containing the polypeptide ^153^QELNEE^158^ (Fig. 3D), indicating that the polypeptide ^153^QELNEE^158^ was the smallest linear epitope required to bind the monoclonal antibodies.

### Character analysis of the neutralizing epitope

**Fig. 4:**
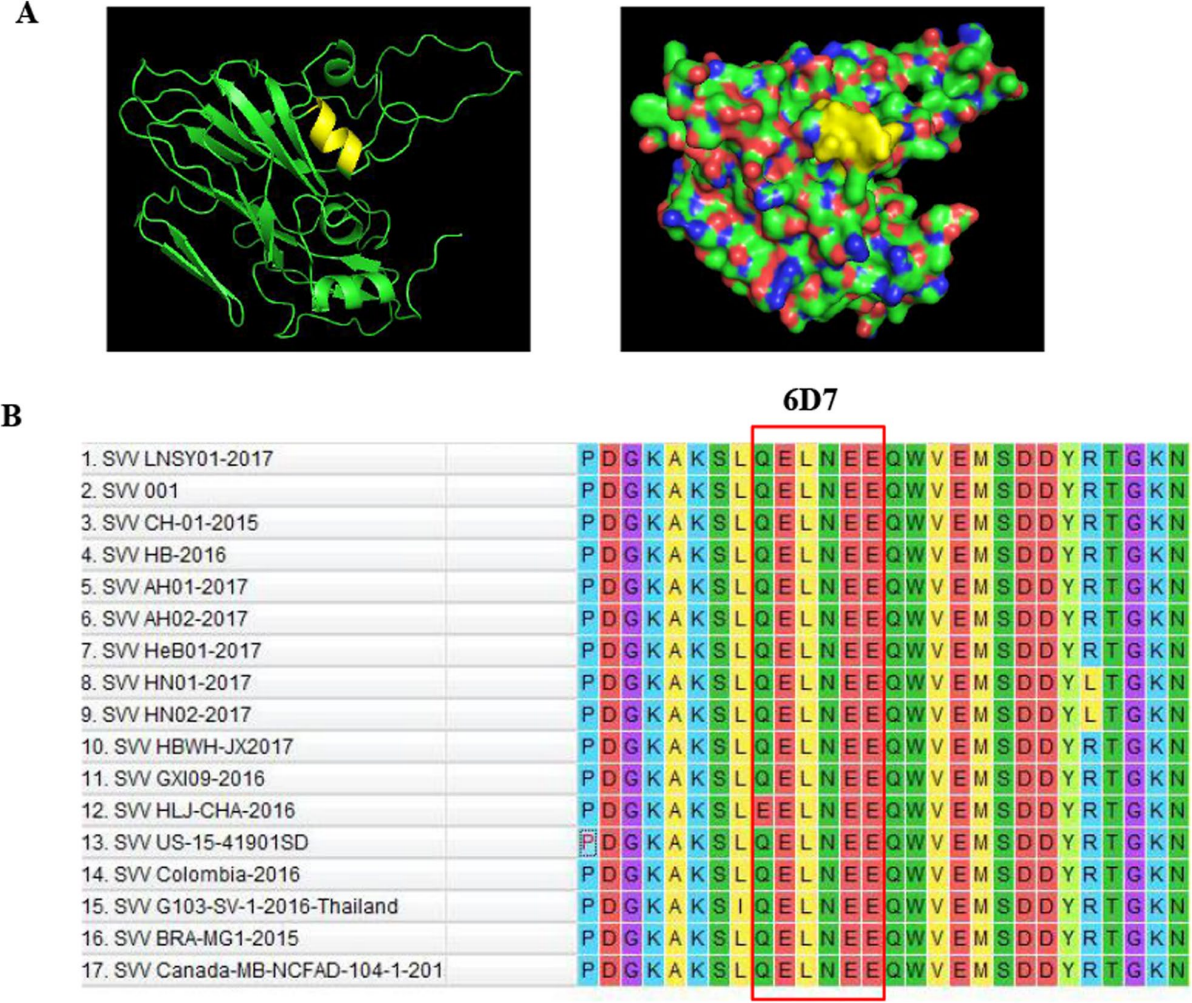
Character analysis of the neutralizing epitope. **A** The relative spatial position of the identified neutralizing epitope is presented in cartoons and spheres from a partially predicted 3D structure of SVV VP2. The epitope recognized by 6D7 is shown in yellow. **B** The amino acid alignment was performed on different SVV isolates VP2 protein using MEGA 7.0.26 software. The smallest linear epitope of ^153^QELNEE^158^ is shown inside the red box

To understand the structural mechanism of the epitope identified by the mAbs, the 3D structure was predicted using an online computer software program. The results revealed that the mAbs recognized neutralizing epitope is fully exposed on the surface of the predicted SVV VP2 structure (Fig. 4A). Sequence alignment analysis of novel epitopes from the different SVV isolates was performed to determine if the novel epitopes identified by the five neutralizing antibodies were conserved among different SVV reference strains. The results showed that the epitope ^153^QELNEE^158^ was conserved in SVV VP2 (Fig. 4B).

### Identification of the pivotal point in the neutralizing epitope

**Fig. 5:**
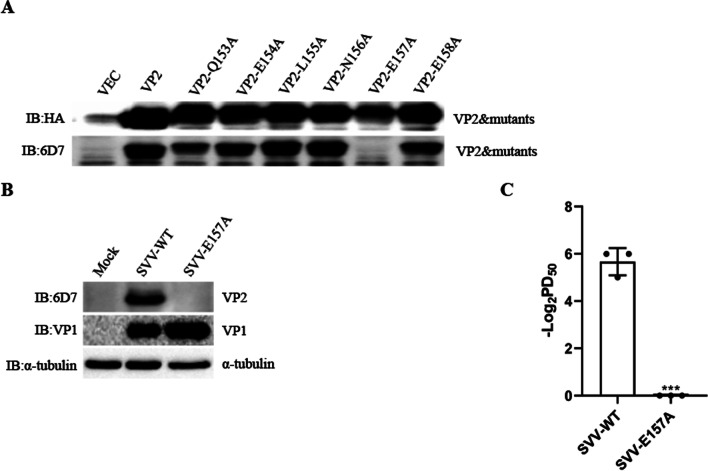
Identification of the pivotal point in the neutralizing epitope. **A** Reactivity of monoclonal antibodies to VP2 mutants. **B** 293 T cells were infected with recombinant virus (SVV-E157A) or wild-type SVV (SVV-WT) for 8 h, and then the cells were collected for western blotting. **C** The neutralizing titers of 6D7-mAbs to SVV-E157A and SVV-WT

Eukaryotic recombinant expression plasmids with single-site mutants of VP2 were constructed, and recombinant proteins were obtained by transfection of 293 T cells. Western blot analysis showed that VP2 protein with a single-site mutant E157A did not react with 6D7-mAb, indicating that E157 was the pivotal point required for VP2 protein to bind the antibodies (Fig. 5A). Additionally, the recombinant virus containing the mutant E157A was constructed to evaluate whether E157A was the key neutralizing point. The virus was serially passaged five times on BHK-21 cells. Stocks from each passage were stored at -80˚C. Each passage viruses were sequenced to identify the stability of the virus. As shown in Fig. 5B, VP1 protein in both WT virus- and E157A mutant virus-infected cells can be detected, but only WT-virus VP2 protein can be detected when the primary antibody was 6D7-mAb. In addition, 6D7-mAb exhibited powerful neutralizing effects on wild-type SVV, but it showed no neutralizing effects on recombinant SVV (SVV-E157A) (Fig. 5C). Taken together, E157 is the pivotal point for 6D7-mAb to neutralize SVV.

## Discussion

Although SVV was successfully isolated and identified in 2002 [1], SVV infection was found to cause herd disease for the first time when there was an outbreak of the porcine idiopathic vesicular disease in 2007 [20]. The same year, clinical signs of vesicular disease appeared in a herd of pigs at a slaughterhouse in the United States. Pig are often co-infected with SVV and other vesicular viruses [19], and the clinical symptoms between SVV and other vesicular viruses are difficult to distinguish [1, 8]. Therefore, the development of monoclonal antibodies is undoubtedly important for the establishment of immunological detection methods. Additionally, neutralizing antibodies can be used to treat viral infections. Due to its oncolytic effect, SVV was often used to explore human cancer treatment [14, 21, 22]. Nevertheless, neutralizing antibodies induced by SVV impaired its oncolytic effect. The construction of a recombinant virus with neutralizing epitope mutations contributes to SVV oncolytic effects.

The capsid protein of SVV is composed of four structural proteins: VP1, VP2, VP3, and VP4, which contain the main epitope region. Dvorak used a modified prokaryotic expression vector pET-24b to express and then purified VP1, VP2, and VP3 proteins and coated the three recombinant proteins with ELISA plates, respectively [18]. Through the verification of a large number of clinical samples, it was found that the sensitivity and specificity of the ELISA plate coated with the VP2 protein alone were better than that of the ELISA plate coated with the VP1 and VP3 proteins. And Goolia used cELISA, VNT, and IFAT to continuously detect antibodies in SVV-infected pig serum [17]. The diagnostic specificity and sensitivity for cELISA were 98.2% and 96.9%, and for VNT were 99.6% and 98.2%. When comparing based on the Kappa coefficient, there is a strong agreement between cELISA, VNT, and IFAT. Virus ultra-high-speed centrifugation is an effective method to obtain high concentration and high purity antigen. In 2017, Mike Strauss used the ultra-ionization purification method and obtained high purity virus particles through a cesium chloride gradient. Three structural proteins VP1, VP2, and VP3 can be seen by SDS-PAGE detection. To develop specific tools to assist SVV detection, we prepared high-purity SVV virus particles and generated 5 monoclonal antibodies against neutralizing epitopes. These five antibodies all recognize the linear epitopes ^153^QELNEE^158^ of VP2 protein, which is conserved among SVV. We also construct the recombinant virus and further demonstrate that E157 is the pivotal point for 6D7-mAb to neutralize SVV. After a comprehensive analysis of the experimental results of Goolia and Dvorak, we speculate that VP2 is likely to be the major immunogenic protein.

Functional analysis showed that all five mAbs can be used for ELISA, western blot, and IFA assays. In addition, the SVV neutralizing activity of the five mAbs was determined by neutralization assay, and the highest neutralizing titer reached 1: 2^10^. In conclusion, we have prepared five neutralizing monoclonal antibodies against SVV VP2 protein to facilitate the early detection of SVV infection using ELISA, western blot, and IFA.

## Conclusions

In the present study, we obtain five SVV neutralizing monoclonal antibodies and identify that E157 is the key amino acid site of the VP2 neutralizing epitope. The five monoclonal antibodies and identified epitopes may contribute to further research on the structure and function of VP2 and the development of diagnostic methods for detecting different SVV strains. Additionally, the epitope recognized by monoclonal antibodies against VP2 protein may provide insights for novel SVV vaccines and oncolytic viruses development.

## Data Availability

All data generated or analyzed in this study are included in this published article.

## Abbreviations

SVV: Seneca Valley virus
ORF: Open reading frame
iELISA: Indirect enzyme-linked immunosorbent assay
IFA: Indirect immunofluorescence assay
CPE: Cytopathic effect
MOI: Multiplicity of infection
WT: Wild type
